# Detection of high mobility group A2 specific mRNA in the plasma of patients affected by epithelial ovarian cancer

**DOI:** 10.18632/oncotarget.2896

**Published:** 2015-02-12

**Authors:** Francesca Galdiero, Annunciata Romano, Rosa Pasquinelli, Sandro Pignata, Stefano Greggi, Emilia Vuttariello, Anna Maria Bello, Celeste Calise, Cono Scaffa, Carmela Pisano, Nunzia Simona Losito, Alfredo Fusco, Daniela Califano, Gennaro Chiappetta

**Affiliations:** ^1^ Genomica Funzionale, Istituto Nazionale per lo Studio e la Cura dei Tumori “Fondazione Giovanni Pascale”, IRCCS, Naples, Italy; ^2^ Dipartimento di Oncologia Uroginecologica, Istituto Nazionale per lo Studio e la Cura dei Tumori “Fondazione Giovanni Pascale”, IRCCS, Naples, Italy; ^3^ Anatomia Patologica, Istituto Nazionale per lo Studio e la Cura dei Tumori “Fondazione Giovanni Pascale”, IRCCS, Naples, Italy; ^4^ Istituto di Endocrinologia ed Oncologia Sperimentale - CNR Dipartimento di Medicina Molecolare e Biotecnologie Mediche, Università degli Studi di Napoli Federico II, Naples, Italy

**Keywords:** Ovarian Cancer, HMGA2, plasma, circulating RNA

## Abstract

Ovarian cancer is the most lethal gynecological malignancy and the high mortality rate is associated with advanced-stage disease at the time of the diagnosis. In order to find new tools to make diagnosis of Epithelial Ovarian Cancer (EOC) at early stages we have analyzed the presence of specific *HMGA2* mRNA in the plasma of patients affected by this neoplasm. HMGA2 overexpression represents a feature of several malignances including ovarian carcinomas. Notably, we detected *HMGA2* specific mRNA in the plasma of 40 out 47 patients with EOC, but not in the plasma of healthy donors. All cases found positive for *HMGA2* mRNA in the plasma showed HMGA2 protein expression in EOC tissues.

Therefore, on the basis of these results, the analysis of circulating *HMGA2* specific mRNA might be considered a very promising tool for the early diagnosis of EOC.

## INTRODUCTION

Ovarian cancer has the highest mortality rate of all gynecologic neoplasms and is the fifth leading cause of female cancer death in western countries [1]. The high mortality rate of Epithelial Ovarian Cancer (EOC) is associated with a late diagnosis because over 70% of women have advanced stage of disease at first diagnosis [2]. Moreover, even after optimal debulking surgery and response to systemic therapy, the risk of recurrence is high and long-term survival remains poor [3].

Therefore, the rapid progression of ovarian cancer and the high mortality rate associated with advanced-stage disease underlines the need of identifying biomarkers that could allow to diagnose the disease in preclinical or presymptomatic phases.

Several studies aimed to detect molecular markers demonstrated that ovarian tumorigenesis is a sophisticated, multifactorial process that involves abnormalities in many gene families including *HMGA2* [4–6].

HMGA2 is a member of architectural chromatin High Mobility Group A (HMGA) protein family. These proteins bind the minor groove of AT-rich DNA sequences through three short basic repeats, called ‘AT-hooks’, and are able to interact with several proteins including various transcription factors. Through these mechanism the HMGA proteins regulate the expression of several genes involved in a wide range of biological processes, such as cell growth, differentiation, apoptosis, and tumorigenesis [7–9]. HMGA2 overexpression has been detected in several human malignancies, in particular pancreatic [10], lung [11], thyroid [12], and ovarian cancer [13, 14] representing a very useful biomarker of malignancy. In particular, we have previously shown that HMGA2 overexpression positively correlated with the body mass index suggesting that the combined evaluation of HMGA2 expression and obesity can be considered a marker of poor prognosis in patients affected by ovarian carcinoma [15].

Previous studies have identified free circulating *HMGA2* mRNA in the plasma/serum of patients affected by breast cancer [16, 17] and leukaemia [18]. Therefore, based on our previous findings that indicated HMGA2 as a promising biomarker for ovarian cancer, the aim of this study has been to investigate whether cell free *HMGA2* mRNA could be detected in the peripheral blood of patients with ovarian cancer.

Here, we report that *HMGA2* specific mRNA was found in 85.1% of the plasma from ovarian cancer patients, but not in the healthy donors, and its detection correlates with the expression of HMGA2 protein in the ovarian carcinoma sections of the same patients.

Therefore, these results allow us to propose the detection of circulating *HMGA2* mRNA as a valid non-invasive tool for the early diagnosis of ovarian cancer.

## RESULTS

### *HMGA2* mRNA was detected in the plasma of EOC patients but not in that of the healthy donors

**Figure 1:**
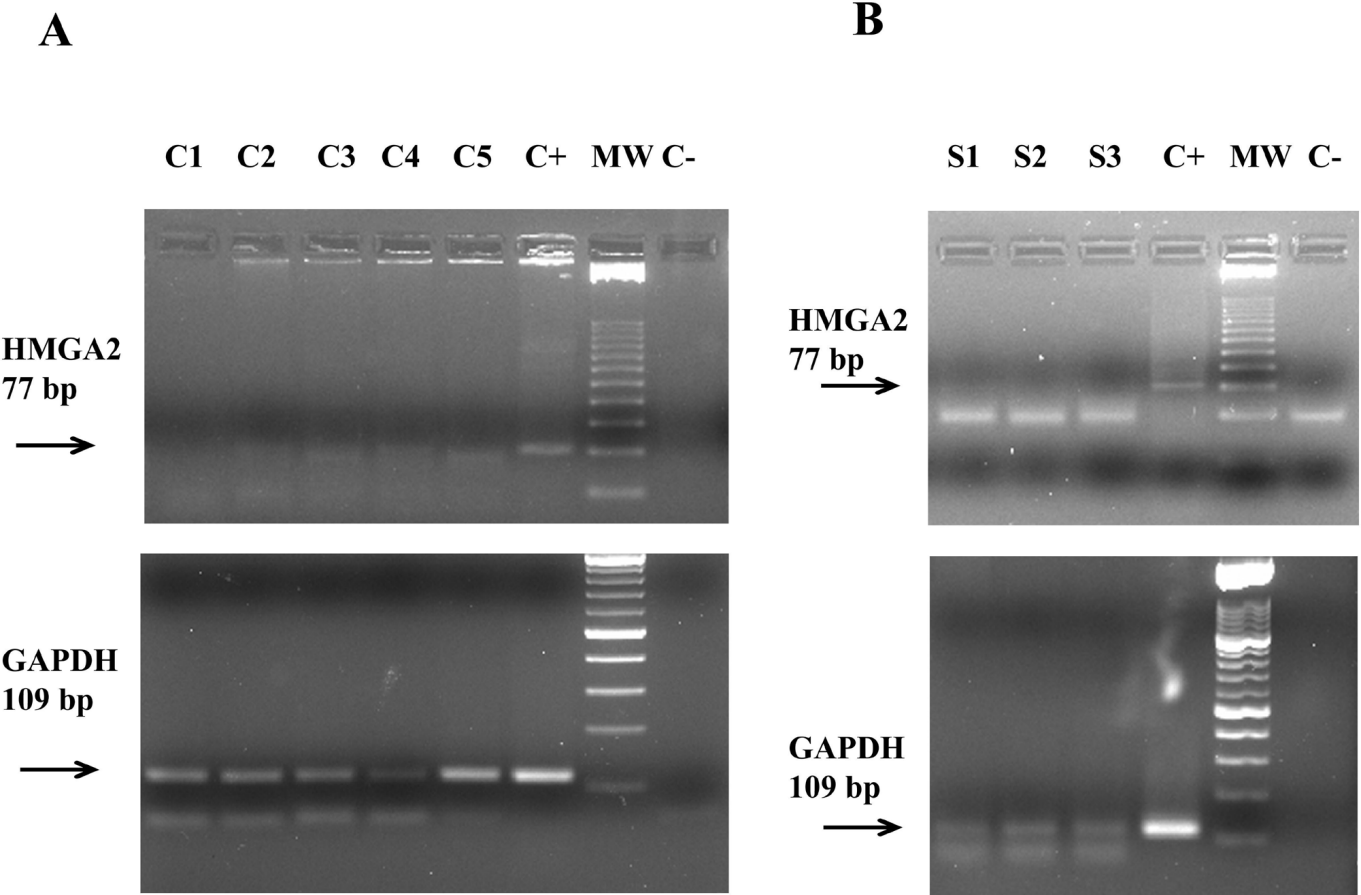
RT-PCR analysis of the *HMGA2* mRNA expression in plasma through 77 bp fragment electrophoresed on a 6% high strength agarose gel In Panel **(A)** five patients with EOC. Lane C1: negative patient; lanes C2–C5: positive patients; lane C+: OVCAR4 cells (positive control); lane MW: molecular weight from 25 bp; lane C−: no template control. The figure below shows the expression of an 109 bp fragment of the housekeeping gene *GAPDH* electrophoresed on a 2% agarose gel. In Panel **(B)** three healthy donors. Lane S1–S3: negative healthy donors; lane C+: OVCAR4 cells (positive control); lane MW: molecular weight from 25 bp; lane C−: no template control. The figure below shows the expression of an 109 bp fragment of the housekeeping gene *GAPDH* electrophoresed on a 2% agarose gel.

**Figure 2:**
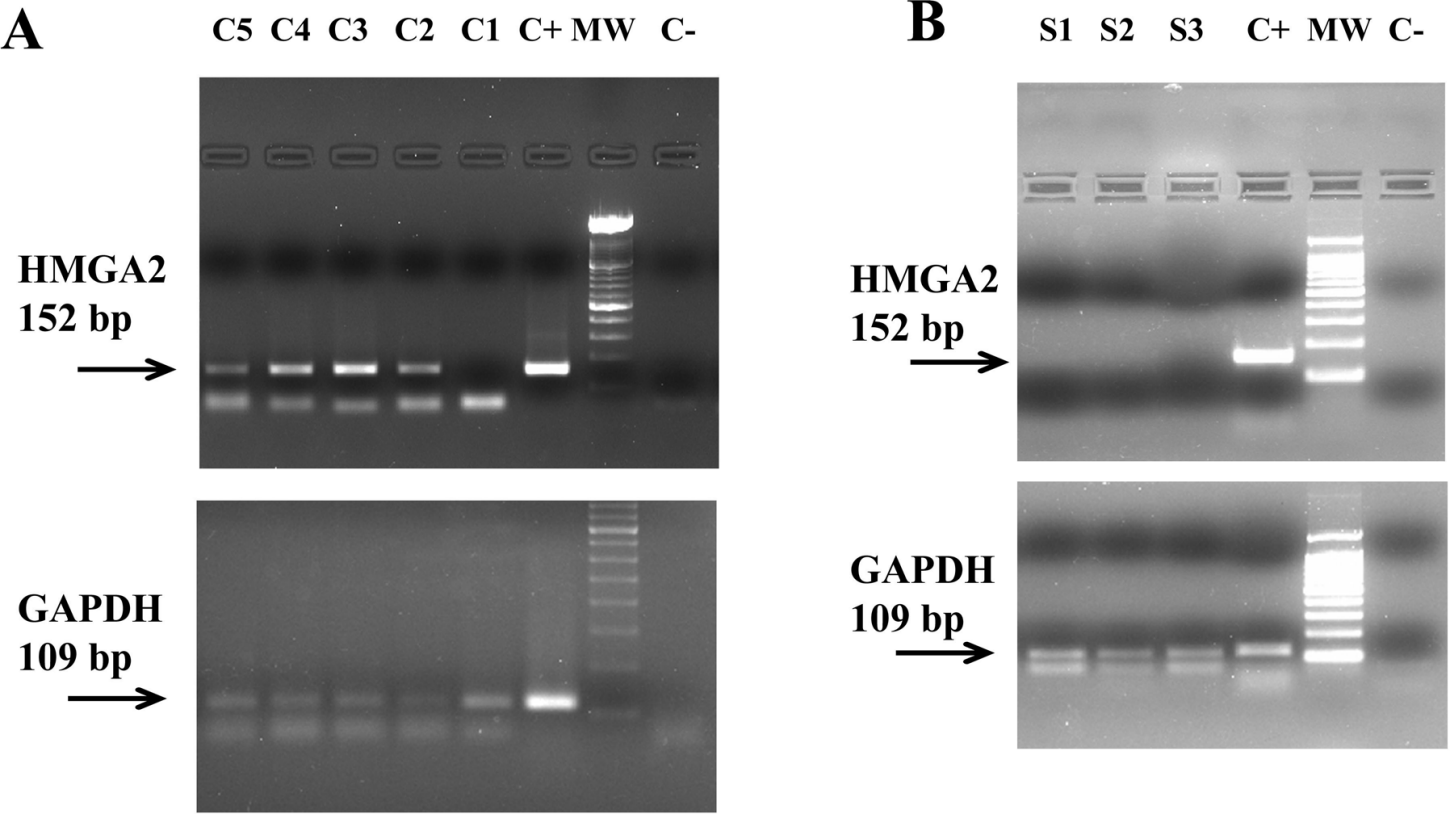
RT-PCR analysis of the *HMGA2* mRNA expression in plasma through 152 bp fragment electrophoresed on a 2% agarose gel In Panel **(A)** five patients with EOC. Lanes C5-C2: positive patients; Lane C1: negative patient; lane C+: OVCAR4 cells (positive control); lane MW: molecular weight from 100 bp; lane C−: no template control. The figure below shows the expression of an 109 bp fragment of the housekeeping gene *GAPDH* electrophoresed on a 2% agarose gel. In Panel **(B)** three healthy donors. Lane S1–S3: negative healthy donors; lane C+: OVCAR4 cells (positive control); lane MW: molecular weight from 100 bp; lane C−: no template control. The figure below shows the expression of an 109 bp fragment of the housekeeping gene *GAPDH* electrophoresed on a 2% agarose gel.

We first analysed the expression of the *GAPDH* housekeeping gene by RT-PCR in the plasma of the ovarian cancer patients. As shown in Figures 1–2, *GAPDH*-specific 109 bp amplicons was detected. Then, we evaluated *HMGA2* mRNA presence in the plasma of 47 patients and 23 healthy donors. The clinical features of the recruited patients are summarized in Table 1.

**Table 1:** Characteristics of ovarian cancer patients

**Age at diagnosis (years)**	
Median	51
Range	29–78
**Stage**	
I/II	11
III	30
IV	6
**Grading**	
Well/Moderate	5
High	32
**Histology**	
Serous	18
Non-serous	26

First, RT-PCR was performed using a primer pair that amplified a 77 bp-fragment spanning parts of the first and second exons of *HMGA2*. Representative results of this analysis in EOC patients and healthy donors are shown in the Figures 1A and 1B, respectively. A faint, but specific band, corresponding to the 77 bp *HMGA2* fragment was detected in the plasma of 40 out of 47 patients but in none of the healthy controls.

Given that the high RNA fragmentation determines, as a consequence, that different regions of the same transcript might be represented differentially, we investigated another region of same gene. In particular, we designed a primer pair to amplify a 152 bp fragment corresponding to a region of the fourth *HMGA2* exon. Representative results are shown in Figure 2A, for cancer samples, and in Figure 2B, for healthy samples. The results obtained with both primer pairs are perfectly overlapping and they are summarized in Table 2. Overall, 40 out of 47 (85.1%) of patients with EOC resulted to be positive for *HMGA2* mRNA expression in peripheral blood.

**Table 2:** Plasma samples from patients with epithelial ovarian cancer and healthy donors analyzed by RT-PCR

Plasma Sample Number	*HMGA2* −	*HMGA2* +
**Ovarian Cancers** (n. 47)	7 (14.9%)	40 (85.1%)
**Healthy donors** (n. 23)	23 (100%)	0

### Detection of *HMGA2* mRNA in the plasma of EOC patients correlates with the protein intratumoral expression

**Figure 3:**
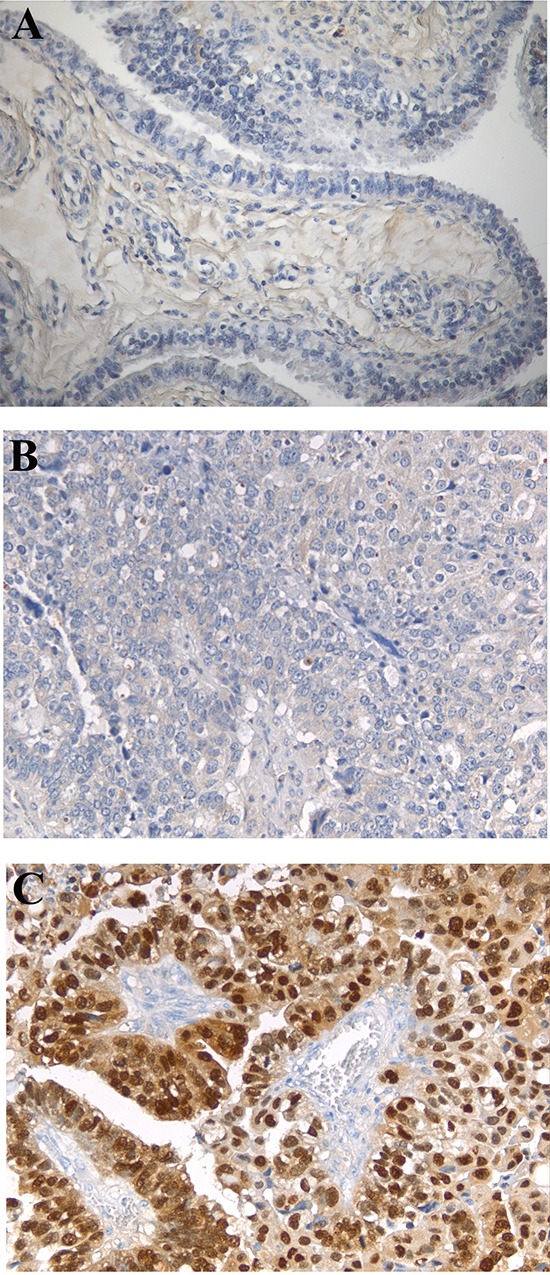
HMGA2 immunohistochemical analysis Negative HMGA2 protein expression in fallopian tube tissue and normal epithelium, with low magnification (200x) **(A)**; two representative cases of serous papillary adenocarcinoma, with low magnification (200x), showing negative **(B)** and positive **(C)** HMGA2 expression.

Subsequently, we analysed the expression of HMGA2 protein in paraffin carcinoma sections from 44 patients out of 47 with EOC, enrolled for *HMGA2* mRNA detection in the plasma, by immunohistochemistry using antibodies raised versus the N-terminal portion of the HMGA2 protein. Three samples could not be evaluated for technical reasons. 91% of the EOC tissues were strongly immunoreactive for HMGA2 (Table 3), whereas no HMGA2 protein was detected in the normal ovarian tissue surrounding the tumor (Figure 3) and in the Fallopian tube tissue with normal epithelium that was used as negative control. As shown in Table 4, all cases found positive for *HMGA2* mRNA in the plasma showed HMGA2 expression at the tumor level. Only 3 out of 7 cases in which we could not detect *HMGA2* mRNA in the plasma were positive for HMGA2 expression at the tumor level.

**Table 3:** Tissue samples of epithelial ovarian cancer analyzed by immunohistochemistry

Number of patients	HMGA2 −	HMGA2 +
**Ovarian Cancer (n. 44)**	4 (9%)	40 (91%)

**Table 4:** Correlations between the expression of *HMGA2* mRNA in peripheral blood and the protein in ovarian cancer tissues

		HMGA2 protein expression	
		Positive (n. 40)	Negative (n. 4)
***HMGA2* mRNA expression**	Positive (n. 37)	37	0
	Negative (n. 7)	3	4

*p* < 0.001

Therefore, these results support the potential use of *HMGA2* mRNA detection in the plasma as a promising diagnostic biomarker for EOC.

## DISCUSSION

The aim of our study has been to evaluate the expression of circulating *HMGA2* mRNA in the plasma of patients affected by EOC in order to have a new tool for the early diagnosis of ovarian carcinoma. Indeed, it is known that HMGA2 is abundantly expressed in ovarian carcinomas [5] but not in normal ovary tissue. Moreover, even though HMGA2 is highly expressed during embryogenesis [20, 21], it is not expressed in adult tissues [22], whereas its expression is abundant in several human malignant neoplasias [23, 24]. Therefore, the detection of *HMGA2* specific mRNA in peripheral blood may indicate the presence of cancer cells.

First, we have developed an extraction protocol in the attempt to obtain a good yield using very small amounts of plasma, since low RNA quantity, high molecular fragmentation and degradation are a major hurdle in the analysis of extracellular RNA and the extraction of nucleic acids from plasma requires copious amounts of starting plasma. Subsequently, we investigated *HMGA2* mRNA expression through RT-PCR. We designed two pairs of primers mapping into distinct regions of the *HMGA2* gene, because of high RNA fragmentation. One pair of primers amplifies a region between the first and second exon common to both known variants of *HMGA2*, the other pair amplifies a fragment of the fourth exon of the variant 2 of *HMGA2* lacking the 3′UTR.

Despite RNA fragmentation, the results obtained by analysing the expression of the fourth exon of *HMGA2* variant 2 were perfectly consistent with those obtained by amplifying the fragment common to both variants of the gene and showed the detection of *HMGA2* mRNA in the plasma of the vast majority (85.1%) of patients with EOC, but not in the plasma of the healthy donors.

Subsequently, we assessed the expression of the HMGA2 protein, by immunohistochemistry, in the tumor sections and their normal counterparts obtained from the same patients enrolled for blood sampling. Consistent with previous reports [13], 91% of the EOC were immunoreactive for HMGA2. Remarkably, in 93% of the patients analyzed the *HMGA2* mRNA presence in peripheral blood correlated with the protein expression in the tumor tissue (Table 4). Only in 3 cases (7%) HMGA2 protein expression in the cancer tissue did not parallel mRNA expression in the peripheral blood. This discrepancy might be due to low HMGA2 protein expression in the cancer tissue, and, then, low amount of circulating *HMGA2*.

Therefore, the detection of circulating *HMGA2* specific mRNA might be an excellent tool for the diagnosis and the monitoring of patients affected by EOC. Indeed, this method is a non-invasive, because it requires only a peripheral blood sample, cost effective, very simple and can also be performed long after a collection, then, allowing large-scale retrospective studies and, therefore, could make possible to screen populations at risk for asymptomatic diseases such as EOC.

## MATERIAL AND METHODS

### Sample collection and blood processing

We collected peripheral blood samples before resection from a cohort of 47 patients with EOC (see Table 1), and from 23 healthy donors. Patients were enrolled into the study between May 2008 until December 2011. Informed consent for the scientific use of biological material was obtained from all patients. All patients were clinically staged according to FIGO (International Federation of Gynecology and Obstetrics) criteria. Patient diagnosis was made according to the WHO 2003 (World Health Organization) classification [19].

### RNA isolation from plasma

After collection EDTA blood samples (5 ml) were immediately placed on ice. Blood samples were centrifuged twice at 1700 rpm for 20 min at 4°C and plasma was collected in cryovials for storage at −80°C until use. After thawing an 75 μl aliquot of plasma was added to 2 ml of TriReagent (Sigma Aldrich) and passed through a 20 gauge-needle for about 10 times. Then, proteinase K (Qiagen) was added to each sample with a final concentration of 0.05 mg/ml and samples were digested for 45 min at 56°C. Following incubation, each sample was passed through a 20 gauge-needle for about 10 times and centrifuged at 4000 rpm for 10 minutes at 4°C.

RNA extraction was then performed through the Qiagen RNeasy mini kit according to the manufacturer instructions. RNA was eluted in 30 μl of RNase free water.

To assess RNA integrity 1 μl of total RNA was analyzed through the Agilent 2100 Bioanalyzer and the RNA 6000 Pico LabChip, according to the manufacturer instruction. Electropherograms showed trace of RNA samples that could not be quantified, characterized by high fragmentation.

### Primer design for RT-PCR

All primers for PCR amplification were designed through the Primer3 tool (http://biotools.umassmed.edu/bioapps/primer3_www.cgi). Primers sequences, together with amplicon size, are listed in Supplementary Table 1. Target gene sequences amplified by the primer pairs were evaluated with the BLAST software in order to check for secondary structures at the site of primer binding. Specificity of PCR products was checked through gel electrophoresis and DNA sequencing.

### PCR amplification and product analysis

First, to eliminate possible genomic DNA contamination, 12 μl of RNA template was digested with DNase (Qiagen) for 10 min at 42°C and then used for the RT-PCR reaction. cDNA was synthesized using the Quantitect ReverseTrascription Kit (Qiagen) according to the manufacturer instruction. Next, we performed a PCR for the *GAPDH* gene (NM_002046.4), as an endogenous control, with the primer pair shown in Supplementary Table 1, at the following conditions: 94°C 2 min, 94°C 20 sec, 60°C 10 sec, 65°C 30 sec (40 cycles) and 65°C 2 min. The 109 bp PCR product was analysed through ethidium bromide staining following electrophoresis on a 2% agarose gel.

The *HMGA2* mRNA (NM_003483.4; NM_003484.1) expression was determined using two pairs of primers (Supplementary Table 1) designed to amplify different gene regions. The first primer pair was used at the following conditions: 95°C 2 min, 95°C 20 sec, 60°C 1 min, 60°C 1 min (40 cycles). The 77 bp product obtained, was subjected to electrophoresis on a 6% high strength agarose gel (GeneAmp, Applied Biosystem) and stained with ethidium bromide. The other primer pair for *HMGA2* gene was used at the following conditions: 94°C 2 min, 94°C 20 sec, 55.2°C 10 sec, 60°C 25 sec (40 cycles). The 152 bp product obtained, was subjected to electrophoresis on a 2% agarose gel and stained with ethidium bromide.

### Sequencing of PCR products

The amplified *HMGA2* products were extracted and purified using the QIAquick Gel Extraction Kit (Qiagen) and sequenced through the Applied Biosystems 3730 DNA Analyzer Apparatus at the Molecular Biology Service (SBM) of the Stazione Zoologica “A. Dohrn” in Naples. Sequences were automatically aligned using ClustalW and hand-checked through the Bio Edit Software v. 7.0.5.3.

### Immunohistochemistry

All analyses were performed on cancerous ovarian tissue and its normal counterpart, which were resected from patients at time of surgery and blood collection. Informed consent for the scientific use of biological material was obtained from all patients. 4 μm thick sections from paraffin-included ovarian cancer specimens were deparaffinised, cleared and antigens were retrieved by microwave irradiation. Slides were treated with a 3% solution of hydrogen peroxidase in methanol to block the endogenous peroxidase activity and then washed in a phosphate buffer solution before immunoperoxidase staining. Slides were then incubated at 4°C overnight with the primary antibody against HMGA2 (anti-rabbit, BioCheck, INC.) diluted 1:300 in a phosphate buffer solution. In the negative controls, the primary antibody was either omitted and replaced with pre-immune serum, or sections were pre-incubated with the HMGA2 control peptide before being challenged with the anti-HMGA2-specific antibody. Tissue sections were incubated with biotinylated anti-rabbit immunoglobulins, and then stained with streptavidin labelled with peroxidase; the signal was developed by using DAB the chromogen as substrate. After chromogen development, slides were washed, dehydrated with alcohol and xylene and mounted with cover slips using a permanent mounting medium.

### Statistical analysis

The statistical comparisons across groups were performed using Fisher's exact test. Statistical significance was established at *p* ≤ 0.01.

## SUPPLEMENTARY TABLE


