# Supplementary material for: Detection of high mobility group A2 specific mRNA in the plasma of patients affected by epithelial ovarian cancer
Source: Oncotarget. 2015 Feb 12;6(22):19328–35. doi: 10.18632/oncotarget.2896 (PMC4662494; doi:10.18632/oncotarget.2896)
Supplement: Supplementary file 1 [file oncotarget-06-19328-s001.pdf]

SUPPLEMENTARY TABLE

Supplementary Table 1: Characteristics of the primers pairs

Acronym	Gene Name	Acc. No.	Primer Forward (5′-3′)	Primer Reverse (5′-3′)	Amplicon size
HMGA2	High mobility group A2	NM_003483.4<br>NM_003484.1	5′ GCTCAGAA GAGAGGAC 3′	5′GGTCTCTTAGG AGAGGGCTCA 3′	77 bp
HMGA2	High mobility group A2	NM_003484.1	5′ATCTACTACC AAGAACCA3′	5′ ACACATAAGG CTCATAGA 3′	152 bp
GAPDH	Glyceraldehyde-3-phosphate	NM_002046.4	5′CCCTTCATTGA CCTCAACTACATG3′	5′TGGGATTTCCA TTGATGACAAGC 3′	109 bp
